# Embryonic development of a centralised brain in coleoid cephalopods

**DOI:** 10.1186/s13064-024-00186-2

**Published:** 2024-06-21

**Authors:** Ali M. Elagoz, Marie Van Dijck, Mark Lassnig, Eve Seuntjens

**Affiliations:** 1 Laboratory of Developmental Neurobiology, Department of Biology, KU Leuven, Leuven, Belgium; 2 Leuven Brain Institute, KU Leuven, Leuven, Belgium; 3 Leuven Institute for Single Cell Omics, KU Leuven, Leuven, Belgium

**Keywords:** Coleoid Cephalopods, Embryogenesis, Neurogenesis, Neuronal Migration, Neural Cell Types, Invertebrate Neurogenesis, Evo-devo

## Abstract

The last common ancestor of cephalopods and vertebrates lived about 580 million years ago, yet coleoid cephalopods, comprising squid, cuttlefish and octopus, have evolved an extraordinary behavioural repertoire that includes learned behaviour and tool utilization. These animals also developed innovative advanced defence mechanisms such as camouflage and ink release. They have evolved unique life cycles and possess the largest invertebrate nervous systems. Thus, studying coleoid cephalopods provides a unique opportunity to gain insights into the evolution and development of large centralised nervous systems. As non-model species, molecular and genetic tools are still limited. However, significant insights have already been gained to deconvolve embryonic brain development. Even though coleoid cephalopods possess a typical molluscan circumesophageal bauplan for their central nervous system, aspects of its development are reminiscent of processes observed in vertebrates as well, such as long-distance neuronal migration. This review provides an overview of embryonic coleoid cephalopod research focusing on the cellular and molecular aspects of neurogenesis, migration and patterning. Additionally, we summarize recent work on neural cell type diversity in embryonic and hatchling cephalopod brains. We conclude by highlighting gaps in our knowledge and routes for future research.

## Background

Coleoid cephalopods, i.e. cuttlefish, octopuses, and squids, are protostome invertebrates with large nervous systems, a broad behavioural repertoire and camera-type eyes and belong to the Mollusca [1–3]. They have divergent life cycles in line with their habitats that can change over different phases of their lives, and have recently been described as holopelagic (whole life cycle pelagic), holobenthic (entire life cycle benthic), merobenthic (with pelagic paralarval (= direct developing larval) phase before becoming benthic) and meropelagic (with alternating pelagic and benthic phases but no paralarval phase) [4].

**Fig. 1:**
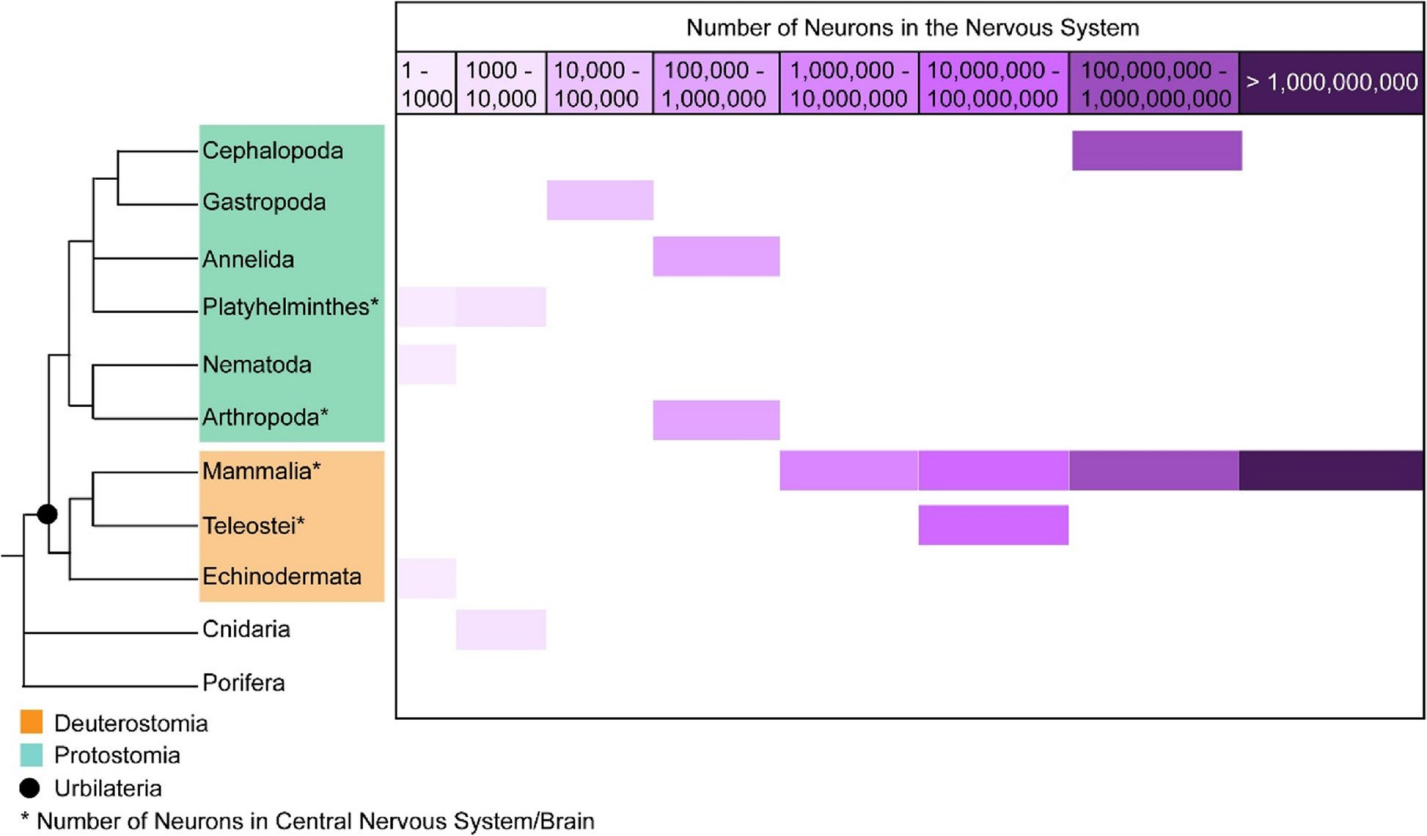
Neural cell count in the nervous system of Bilateria, Cnidaria and Porifera. The total number of neurons in the nervous system (depending on the size of the nervous system and availability of the data) of Bilateria, Cnidaria and Porifera. Bilateria are divided into Protostomia and Deuterostomia. Urbilateria is the last common ancestor between protostomes and deuterostomes. Cephalopods have the highest number of neurons in their nervous system among protostomes. Their neuronal count is even higher than that of some deuterostomes. The mammalian brain is in a neuronal count range similar to the cephalopod nervous system. Porifera has no nervous system. The neuronal count number representatives for the selected groups: Cephalopoda: [5, 6]; Gastropoda: [13]; Annelida: [14, 15]; Platyhelminthes*: [16, 17]; Nematoda: [18]; Arthropoda*: [19]; Mammalia*: [20, 21]; Teleostei*: [22]; Echinodermata: [23]; Cnidaria: [24]; Porifera: [25]. Asterisk (*) symbolises that the number range indicated is for either the brain or the CNS. Figure credits: adapted from figure presented by Jessica Stock at the Urchin (DBSUMI) Conference in October 2023

The cephalopod nervous system comprises a large centralised brain located between the eyes, a neural cord in each arm, and distributed ganglia in the mantle important for body shape control, and control of gastrointestinal function [2, 5]. The total nervous system of the studied adult coleoid species such as octopus is estimated to account for approximately five hundred million neurons, making it the largest of all invertebrates [5, 6]. This neuronal count exceeds that of the other protostomes and some deuterostomes, and is in the same range as that of a medium-sized mammalian brain such as a ferret brain (Fig. 1) [7]. Their extended behavioural repertoire reflects the complex cognitive abilities they have developed throughout their evolution. They can change their skin colour and texture in milliseconds due to the direct control of their chromatophores by the nervous system. They camouflage as a form of communication and defensive mimicry [8]. They use tools like rocks, shells, and other items to cover their bodies and create a mobile den [8]. They have developed novel morphological features compared to other mollusks, such as a eight sucker-lined arms and in addition to that, for decapods, 2 tentacles [9]. Their large and repetitive genome displays innovations such as significant genome reorganisations and specific gene family expansions, for example, within the protocadherin genes and zinc-finger transcription factors, which are thought to play crucial roles during neural development [10, 11]. In addition, they have evolved extensive RNA editing that endows them with immense neural plasticity and the capacity to adapt to changing environmental conditions[11, 12].

Coleoid cephalopods have evolved and expanded their central nervous system independently from mammalian brains, which makes them ideal organisms to investigate the mechanisms of large centralised nervous system evolution from an unconventional perspective [26, 27]. Studying neurogenesis and neural patterning in coleoid cephalopods is a starting point to provide insights into this fundamental question.

Neurogenesis is the process of generating neurons from neural stem cells. This process occurs during embryonic development to develop a nervous system, but in many species, continues later in life as adult neurogenesis [28, 29]. Neurogenesis is regulated by cell-intrinsic factors, such as transcription factors, as well as cell-extrinsic factors, such as secreted signalling molecules, that regulate the process in a precise temporospatial manner. First, neural fate is induced in specific ectodermal epithelial areas of the embryo, followed by the proliferation of neural stem cells. In species with larger-sized nervous systems, neuroepithelial stem cells initially proliferate symmetrically to expand the progenitor pool [30]. According to the canonical view, at the onset of neurogenesis, they switch to divide asymmetrically in order to generate neurons or secondary populations of proliferating progenitors [31]. The specification steps that neural progenitor cells follow varies depending on species and neurogenic region [32–35]. Current evidence from *Drosophila* neuroblast lineages and retinal progenitors in *Xenopus* indicate that the fate of the postmitotic daughter cells gets determined at the moment of the last cell division of the progenitors [36–40]. Different neuronal subtypes are consecutively generated from the same multipotent stem cell by a sequence of temporally regulated intrinsic changes in combination with extrinsic factors. In the *Drosophila* optic lobe, for instance, fate is regulated by the expression of temporally restricted transcription factors, ensuring the timely appearance and organisation of different neuronal subtypes [41, 42]. In the murine cerebral cortex, extrinsic factors from the cerebrospinal fluid as well as feedback factors from the postmitotic neurons regulate sequential generation of different cell types [43, 44]. Timing is also important for the coordination of neurogenesis in the mammalian cortex, where different neuronal layers are formed at specific time windows during corticogenesis. This process is orchestrated by the intrinsic capacity of a gradually depleting multipotent progenitor pool guided by extrinsic cues to generate 8-9 neurons that occupy different laminar positions [45, 46]. Besides time, the spatial location of neurogenic stem cells is an important factor in fate determination. In mammals, pallial and subpallial stem cells give rise to excitatory and inhibitory neurons of the neocortex, respectively [47]. In *Drosophila*, the ventral nerve cord neuroectoderm is patterned along the anterior-posterior axis in neuromeres that generate different neuronal subtypes [48, 49]. After neurogenesis and fate specification, newborn neurons further differentiate, mature and connect with other neurons. In vertebrate brains, newborn neurons migrate away from the neurogenic niche to other brain areas. During this process, they are guided by axons or by cues that are often also used by growing axons to connect more distant brain regions [50, 51]. In addition, during *Drosophila* neurogenesis newborn neurons have been observed to migrate into the optic lobe medulla during the pupal phase [52, 53]. Our current knowledge about the process of neurogenesis in species with larger, centralised nervous systems predominantly comes from model species, such as zebrafish, chicken and mouse, and *Drosophila* [54]. Recent advancements in technological tools, such as next-generation sequencing technologies, molecular biology and biotechnology techniques, and genome editing tools, have led to the investigation of neurogenesis and neural patterning in more species, including coleoid cephalopods [55–57]. Here, we summarise recent findings on neurogenesis, the molecular mechanisms responsible for its regulation and neural cell types in the embryonic coleoid cephalopod central nervous system.

## Anatomical organisation of the coleoid cephalopod central nervous system

The coleoid cephalopod central nervous system (CNS) makes up one-third of the adult nervous system and comprises more than 30 differentiated lobes and around 12 nerve tracts [5, 58]. Each lobe has a central neuropil which contains mainly neurites and glial cells, and is surrounded by a perikaryal layer of neuronal cell bodies [27, 59]. Anatomically, the cephalopod CNS is located in between the eyes and consists of a circumesophageal central brain comprising supraesophageal (SEM) and subesophageal (SUB) masses that are surrounded by cartilaginous tissue and two periesophageal masses (PEM), which are located on each lateral side of the central brain (example of a 3D cephalopod brain atlas in Montague et al., 2023 [58]).

The SEM, located anteriorly to the esophagus, functions as the higher cognitive and motor center and consists of around 12 lobes (variation among species) organised in two major lobe complexes: vertical lobe complex and basal lobe complex. The vertical lobe complex is responsible for learning and memory and is comparable to the mammalian limbic system [60–63]. The basal lobe complex is the brain region mediating the higher motor functions involved in the movement of the body parts, and control of chromatophores and papillae [64, 65].

The SUB, located posteriorly to the esophagus, has intermediate and lower motor functions. It controls breathing, feeding and movements involved in defence mechanisms such as inking and camouflage [66]. The SUB comprises 4 lobe complexes made up of 17 lobes: brachial lobe complex, magnocellular lobe complex, palliovisceral lobe complex and pedal lobe complex. The brachial lobe complex is a motor control center for the arms and feeding. The magnocellular lobe complex is responsible for breathing. The palliovisceral lobe complex controls the locomotion. The pedal lobe complex is responsible for the movement of the body parts and is in control of chromatophores and papillae on the mantle and arms [58, 64].

Each PEM is formed from the optic tract complex, which comprises an optic lobe, a peduncle lobe, a dorsolateral lobe, an olfactory lobe and an optic gland. The optic tract complexes are in charge of anything relevant to visual processing, including visual learning and memory, and visio-motor integration [58, 67–70].

## Neurogenesis and embryonic brain development

### Embryonic brain growth

**Fig. 2:**
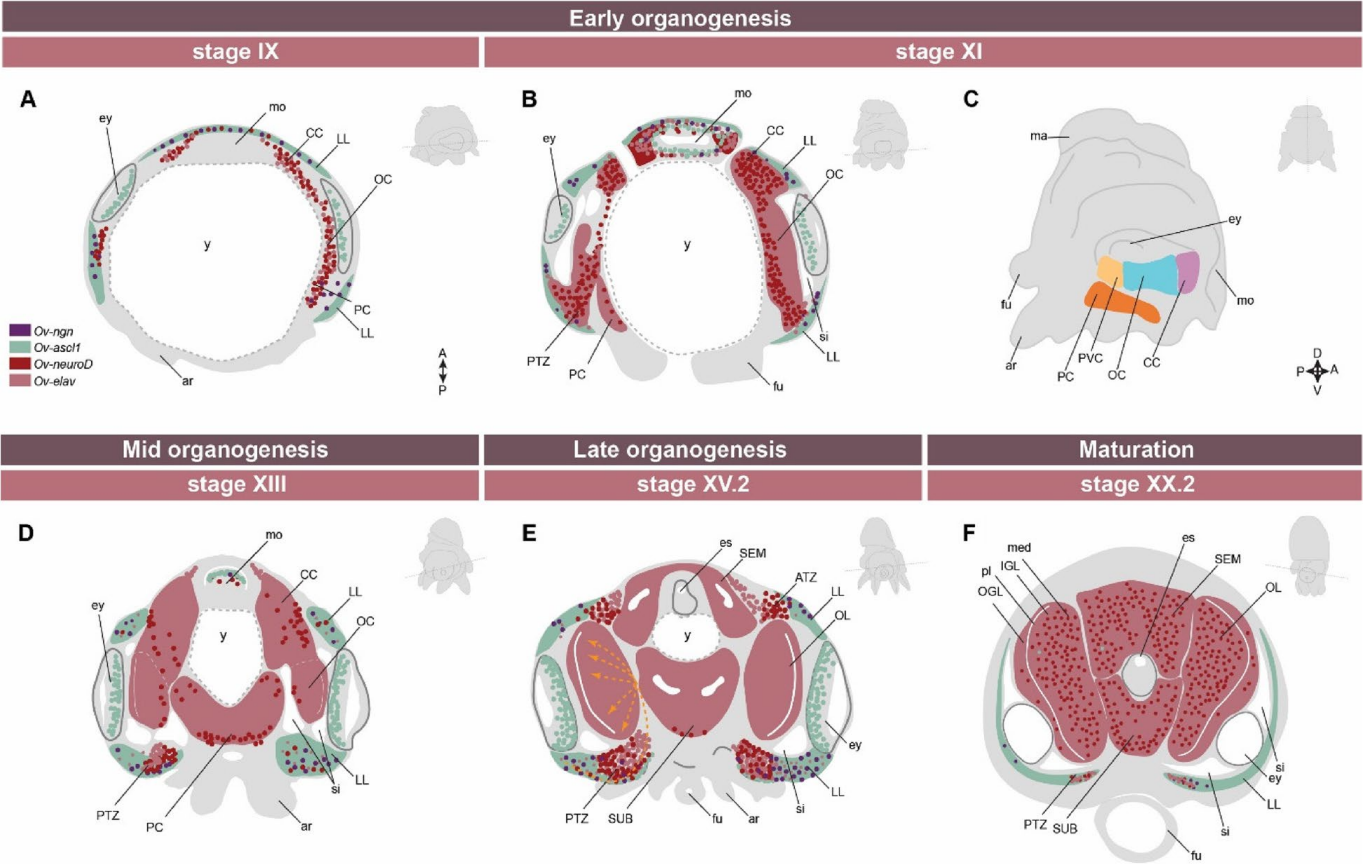
Overview of the expression of neurogenic genes in the cephalopod brain throughout the embryogenesis. **A**., **B., D**.-**F**. Schematic depiction of the expression domains of *Ov-ngn* (purple), *Ov-ascl1* (green), *Ov-neuroD* (red) and *Ov-elav* (pink) from early organogenesis to late pre-hatching phases on horizontal cephalopod (*O. vulgaris*) brain sections. The larger solid color domains indicate high expression level of the specific gene in the majority of the cells located in that region. Points indicate either lower expression in the general region or high expression levels in a few cells. The orange arrows during late organogenesis indicate one of the migratory routes taken by the neural progenitor cells generated in the lateral lips, migrating through the posterior transition zone and entering the brain (based on the lineage tracing experiments). As the brain develops, the lateral lips diminish in size. **C**. Schematic illustration of the brain cords in a cephalopod (*O. vulgaris*) sagittal transection (inside view) during early organogenesis (stage XI). Schematic illustration of the embryo based on the developmental stage (*O. vulgaris)* for each brain section is provided on the top right. The approximate location and position of the section are delineated with a dashed line on the embryo. Abbreviations: A, anterior; ar, arm; ATZ, anterior transition zone; CC, cerebral cord; es, esophagus; ey, eye; fu, funnel; LL, lateral lips; mo, mouth; OC, optic cord; OL, optic lobe; P, posterior; PC, pedal cord; PTZ, posterior transition zone; PVC, palliovisceral cord; si, sinus ophthalmicus; SEM, supraesophageal mass; SUB, subesophageal mass. The embryonic schemes are based on Deryckere et al., 2020 [81]. Temporospatial patterning of the neurogenic genes and scheme illustrating interconnected nature of central brain cords in panel C are reproduced from Deryckere et al., 2021 [79]

Molluscan nervous systems follow either a ganglionic or cordal pattern of embryonic neurogenesis. Conchiferan molluscs, such as bivalves, gastropods and scaphopods develop their nervous systems based on ganglia: dense, well-characterised clusters of neuronal cell bodies surrounding a centralised neuropil [13, 71–73]. In contrast, aculiferan molluscs, such as polyplacophorans, develop a nervous system organised as cords distinguished with a layer of neuronal cell bodies covering longitudinal neuropil [71, 74]. In the 20^th^ and early 21^st^ centuries, researchers hypothesised that coleoid cephalopods also followed a ganglionic pattern of neurogenesis [75–78]. Contrary to this, recent studies have shown that the coleoid cephalopod brain develops from cords [59, 79]. These studies shown that during early embryogenesis, development of extensive, rope-like neurogenic territories is observed during early allocation of the neurons which is common in cordal origin of neurogenesis, instead of nodular clusters which is typical for a ganglionic origin of neurogenesis [59, 79] (Fig. 2). This cordal patterning of neurogenesis is also observed in nautiloid embryos, indicating that multiple cord-based neurogenesis is a conserved origin across Cephalopoda [59, 80].

Early histological studies on the embryonic brain development of squids *Loligo vulgaris, Sepioteuthis lessoniana*, *Todarodes pacificus*, *Idiosepius paradoxus,* and the octopod *Octopus vulgaris* have shown that morphogenesis of coleoid brains is highly similar [75–78, 82, 83]. The development of the cephalopod brain starts with the emergence of the neurogenic placodes during the epiboly stage of the embryo. The neurogenic cerebral, optic, palliovisceral and pedal placodes arise as ectodermal thickenings in the equatorial plane of the embryo [78]. The cerebral placodes emerge laterally to the mouth, the optic placodes arise anteriorly to the eye primordia, and the pedal and palliovisceral placodes are located anteriorly and posteriorly of the statocyst primordia respectively [78]. In the early stages of organogenesis, these ectodermal placodes are internalised, interconnected and longitudinally elongated to form the cordal anlagen of the circumesophageal cephalopod brain [59, 79]. As the brain develops, the earlier generated neurons mature and form the first neuropil. The formation of commissures in the developing cords leads to the formation of lobe complexes within the circumesophageal masses. The supraesophageal mass is formed from the anteriorly positioned cerebral cords, whereas posteriorly positioned palliovisceral and pedal cords join to develop into the subesophageal mass, and the bilaterally located optic cords grow into optic lobes [79].

### Neurogenesis and the brain neurogenic niche

During early organogenesis, after the first cordal structures have been established, the growing embryonic cephalopod brain is predominantly populated by postmitotic, newborn neurons that express the pan-bilaterian neuronal marker *embryonic lethal, abnormal vision* (*elav*) and low to no *synaptotagmin* (a synaptic vesicle protein present in differentiated, mature neurons) [79]. After the primary cords have been established, the cephalopod brain seems to lack dividing neural progenitor cells within the cords. Proliferation, marked by *proliferating cell nuclear antigen* (*pcna*) and phosphohistone H3, is observed more in the paraocular area, previously described as the "Kopflappen" [75] then "Anterior Chamber Organ" [78, 84, 85] or and recently renamed to "Lateral lips" [79]. These neural progenitor cell populations have been identified using evolutionarily conserved proneuronal regulatory markers, such as basic helix-loop-helix (bHLH) transcription factors, *achaete-scute* (*asc*) and *neurogenin* (*neurog*) (Fig. 2) [59, 79]. *SoxB1* (*sox2*), a common marker of early neural progenitors in many species, seems to be expressed beyond the neural progenitor zones, and also in postmitotic neuronal subpopulations, making it a less suitable neural progenitor marker in cephalopods [79, 86]. As neuronal progenitor cells differentiate, they start expressing another conserved bHLH transcription factor known as neuron differentiation marker *neuroD* [59, 79]. The sequential expression of bHLH transcription factors is highly conserved for neurogenesis throughout Bilateria [79]. *neuroD* is predominantly expressed in the areas which interconnect the lateral lips (LL) and brain cords anteriorly and posteriorly in a bow-shaped 3D structure, thus called anterior and posterior transition zones, respectively [87]. During embryonic development, the neural progenitor population at the lateral lips progressively gets consumed, and at hatching, only a small remnant is left (Fig. 2) [79]. Since the adult octopus brain is estimated to have a thousand times more neurons than the brain of the hatchling (200 million to 200,000), extensive neurogenesis must occur post-hatching [27]. The post-hatching source of neural progenitor cells and the process of post-hatching neurogenesis, which is responsible for the neural cell count increase and brain lobe development and maturation is still a mystery to be solved.

**Fig. 3:**
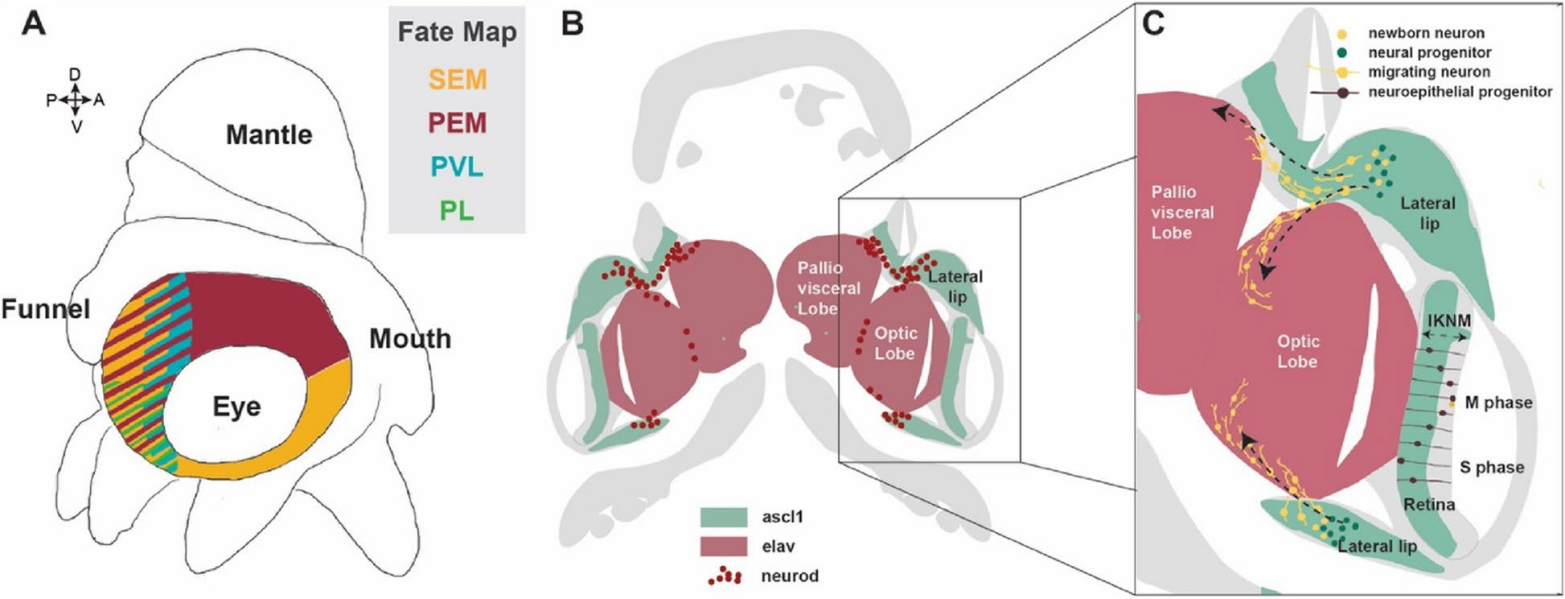
Fate specification and neuronal cell migration in the developing cephalopod brain. **A.** Fate map of the cephalopod lateral lip region (depiction on a Stage XIII *Octopus vulgaris* embryo), based on lineage tracing studies in octopus and squid embryos. Abbreviations: SEM: supra-esophageal mass, PEM: peri-esophageal mass; PVL: palliovisceral lobe; PL: pedal lobe. **B.** Schematic coronal section overview of a developing cephalopod in the organogenesis stage (*O. vulgaris –* Stage XIII). Green areas express *ascl1*, neural progenitor marker, red areas depict postmitotic neuronal *elav* expression, and dark red dots indicate *neuroD* expression. **C.** Cell migration. In the retina, neuroepithelial progenitors (brown) display interkinetic nuclear migration (IKNM): in the M phase, nuclei are at the apical surface where they undergo mitosis, generating a postmitotic cell (yellow) and a progenitor. The nucleus of this progenitor migrates to the basal side, where it spends the S phase. The lateral lip (LL) does not have a pseudostratified epithelial structure, and neurogenesis generates postmitotic neurons (yellow) that migrate away to the brain along different trajectories (dashed arrows)

In contrast to the cephalopod retina [84] and the vertebrate neural tube [88], the lateral lip neurogenic zones are not organised as a pseudostratified neuroepithelium (a single epithelial layer which appears stratified because cell nuclei occupy different apicobasal positions) [89]. The primary, self-renewing progenitors (solely expressing *ascl1*+) appear more laterally, whereas differentiating postmitotic cells (*neuroD*+) are intermingled with neural progenitor cells (*ascl1*+) in the more medial regions, suggesting a different organisation of the neurogenic niche (Figs 2 and 3) [79].

Lineage tracing in *Doryteuthis pealeii* and *Octopus vulgaris* has shown that different areas in the lateral lips contribute cells to different regions of the central brain (Fig. 3A, Table1) [79, 84]. These findings indicate that the progenitor zone is patterned and the destination of neurons is determined while the cells are in the lateral lips. Cephalopods thus follow a similar strategy of spatial patterning of the progenitor zone compared to vertebrates. Deryckere et al. also found preliminary evidence for temporal patterning, as the optic lobe cortex cells were generated proportionally more at early stages than the optic lobe medulla cells, and the palliovisceral lobe could only be traced at earlier stages [79]. More in-depth studies are needed to reveal whether specific cell types are born at specific time points during development.

**Table 1:** Lineage tracing of the neural progenitor cells

**Location in Lateral Lips**	**Destination in brain of the hatchling**
(Ventral)-posterior quadrant (early organogenesis)	Palliovisceral and pedal^a^ lobe (SUB)
Posterior (throughout the embryogenesis)	Inferior and superior frontal lobes SEM
Ventral	Basal lobes SEM
Ventral-anterior	Majority of SEM cells
Dorsal-anterior quadrant	Optic lobes (PEM)
Posterior (early-mid organogenesis)	Optic lobe cortex (PEM)
Posterior (late organogenesis)	Optic lobe medulla (PEM)
Posterior & Ventral	Olfactory & Peduncle lobes (PEM)

^a^Pedal lobe was only traced in Koenig et al. [84] while palliovisceral lobe was only traced in Deryckere et al. [79]

The development and maturation of neurons and processes in the coleoid cephalopod brain during embryogenesis is asynchronous [27, 79, 90]. For instance, at hatching, the optic lobes have a structure with a clearly laminated cortex and a cauliflower-organised medulla reminiscent of the adult optic lobe, while other brain lobes are far less developed than their adult counterpart [75, 78, 79, 91, 92]. Moreover, the cell density and the size of the brain lobes at the end of embryonic neurogenesis also differ across different coleoid species. These variations are linked to the species-specific differences in the life cycle, the embryonic developmental time, egg size, and behaviour repertoire that hatchlings need to possess to survive post-hatching. Thus, relative brain maturity at hatching differs between cephalopod species. For example, many of the brain lobes in *Todarodes pacificus* are immature before hatching, and the hatchlings are known to be suspension feeders and not active predators [76]. At what embryonic stage coordinated neuronal activity arises in the cephalopod brain is also unclear. As an example, a holobenthic cephalopod species, *Sepia officinalis,* shows distinctive signs of visual learning related to food imprinting at least a week before hatching, indicating this axis is already operational during embryonic development [93, 94], whereas another holobenthic cephalopod species *Octopus berrima* does not [95].

### Neuronal migration

Since neurons in the cephalopod brain are not born locally, it was hypothesised that they display migratory behaviour from the lateral lips to their final destination in the brain. Migratory behavior of cephalopod cells was first observed by Marthy and Aroles in *Loligo vulgaris*, where cells seemed to migrate out of "oculo-ganglionar complex" explants containing retina, lateral lips and optic lobe tissue [96]. Because the explant contained different tissues, it was difficult to understand where these cells originated, yet they seemed to obtain bipolar and multipolar neuronal shapes [96]. More recent lineage studies using CFDA-SE in *Octopus vulgaris* embryos labelled cells along a trajectory spanning the lateral lips, posterior transition zone (PTZ) and optic lobe, providing further evidence that after being born, neurons travel over long distances to their final destination [79]. Different routes of migration are delineated by tissue boundaries that arise within the embryo, but molecular guidance cues have not yet been described (Fig. 3B,C). Besides long-distance neuronal migration, nuclear migration has been observed in the cephalopod retina [97]. There, neurogenic progenitors are organised in a typical pseudostratified neuroepithelial layer that generates photoreceptors and supporting cells. Nuclei of neuroepithelial stem cells migrate in a coordinated fashion with the cell cycle, a process known as interkinetic nuclear migration (Fig. 3B,C) [97]. Intriguingly, both long-distance migration and interkinetic nuclear migration are mechanisms occurring during the development of large brain structures such as the mammalian cerebral cortex, suggesting they were adopted independently and might be required for the growth of large nervous systems.

## Molecular mechanisms regulating embryonic brain development

The exploration of the cephalopod neurogenic toolkit started over two decades ago by studying the temporospatial expression patterns of specific intrinsic (cytoplasmic or nuclear factors such as transcription factors and post-transcriptional regulators) and extrinsic (components of signaling pathways located outside of cells) factors.

### Intrinsic factors

Most of these molecular studies have focused on one specific gene family of transcription factors, examining the temporospatial patterning of the identified orthologs of specific genes throughout embryogenesis. In addition, there have been attempts to investigate the neural regionalisation of the brain by studying either a specific region (anterior/posterior part of the brain) or patterning of the neural axis (for instance, along the mediolateral axis discussed in Buresi et al., 2016 [98]). Since the visual system is seen as an extension of the nervous system, the majority of eye development and visual system studies on cephalopods have also provided information about the expression patterns of the investigated genes in the brain. Almost all these studies have been done on squid species (*Idiosepius notoides*, *Idiosepius paradoxus, Sepia officinalis, Euprymna scolopes, Loligo opalescens,* and *Doryteuthis pealeii*). Considering these conditions and to the best of our knowledge, we attempted to provide tables summarising the spatial and temporal expression pattern of the intrinsic factors expressed in CNS studied in coleoid cephalopods over development (Refer to Table 2 for spatial expression of TFs, Table 3 for temporal expression of TFs).

**Table 2:**
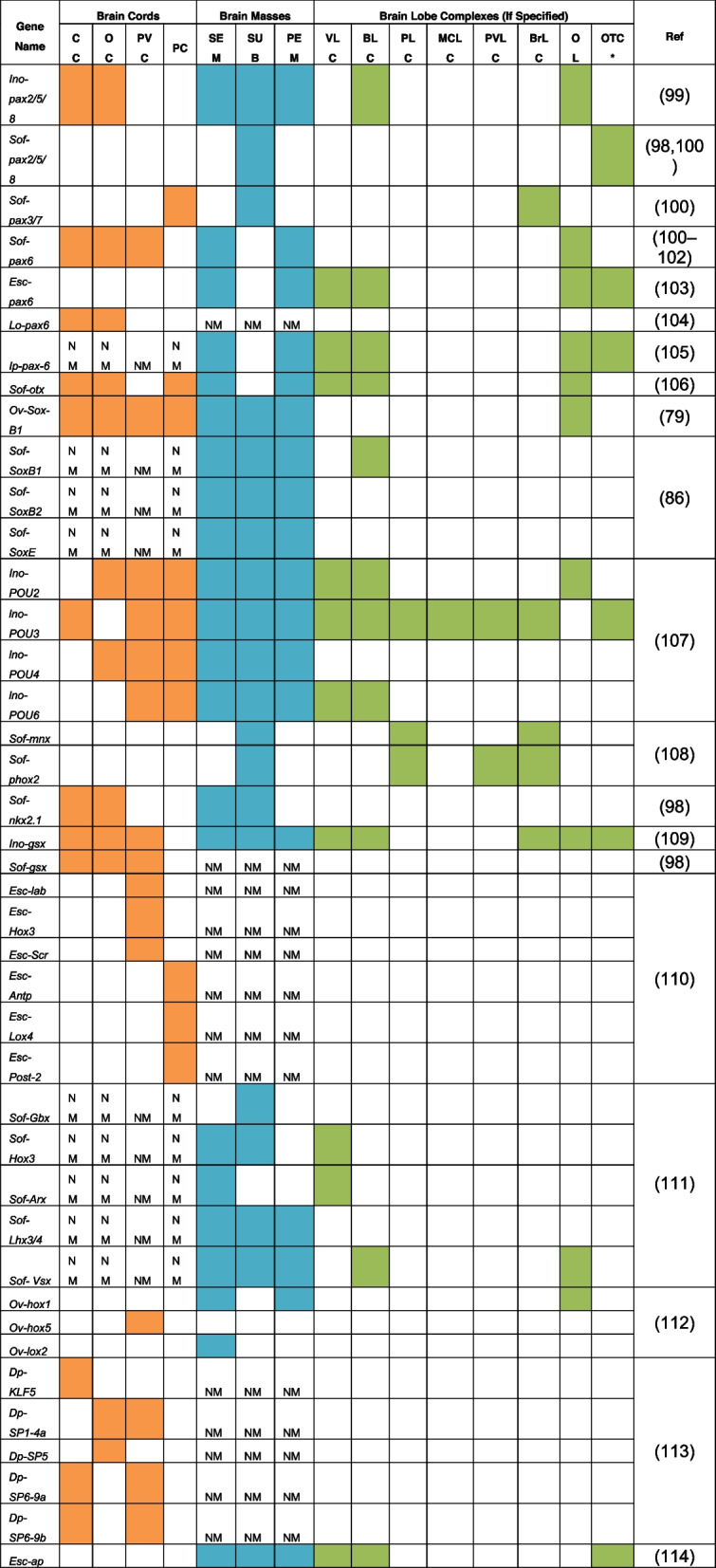
Spatial expression of the transcription factors in the CNS based on ISH

The table summarises the spatial expression pattern of the transcription factors studied on cephalopod species based solely on ISH. The table is divided into 3 main parts: Brain Cords, Brain Masses and Brain Lobe Complexes, if specified in the paper. The expression intensity of the genes is indicated by color: Color - Expression, No Color – No Expression, NM - Not mentioned in the paper. Abbreviations: BLC: Basal Lobe Complex; BrLC: Brachial Lobe Complex; CC: Cerebral Cord; Dp: *Doryteuthis pealeii*; Esc: *Euprymna scolopes*; Ino: *Idiosepius notoides*; Ip: *Idiosepius paradoxus*; Lo: *Loligo opalescens*; MCLC: Magnocellular Lobe Complex; NM: Not Mentioned; Ov: *Octopus vulgaris*; OC: Optic Cord; OL: Optic Lobe; OTC*: Optic Tract Complex except OL; PC: Pedal Cord; PEM: Periesophageal Mass; PLC: Pedal Lobe Complex; PTZ: Posterior Transition Zone; PVC: Palliovisceral Cord; PVLC: Palliovisceral Lobe Complex; Ref: References; Sof: *Sepia officinalis*; SEM: Supraesophageal Mass; SUB: Subesophageal Mass; VLC: Vertical Lobe Complex [99], [98, 100], [100], [100–102], [103], [104], [105], [106], [79], [86], [107], [108], [98], [109], [98], [110], [111], [112], [113], [114]

**Table 3:**
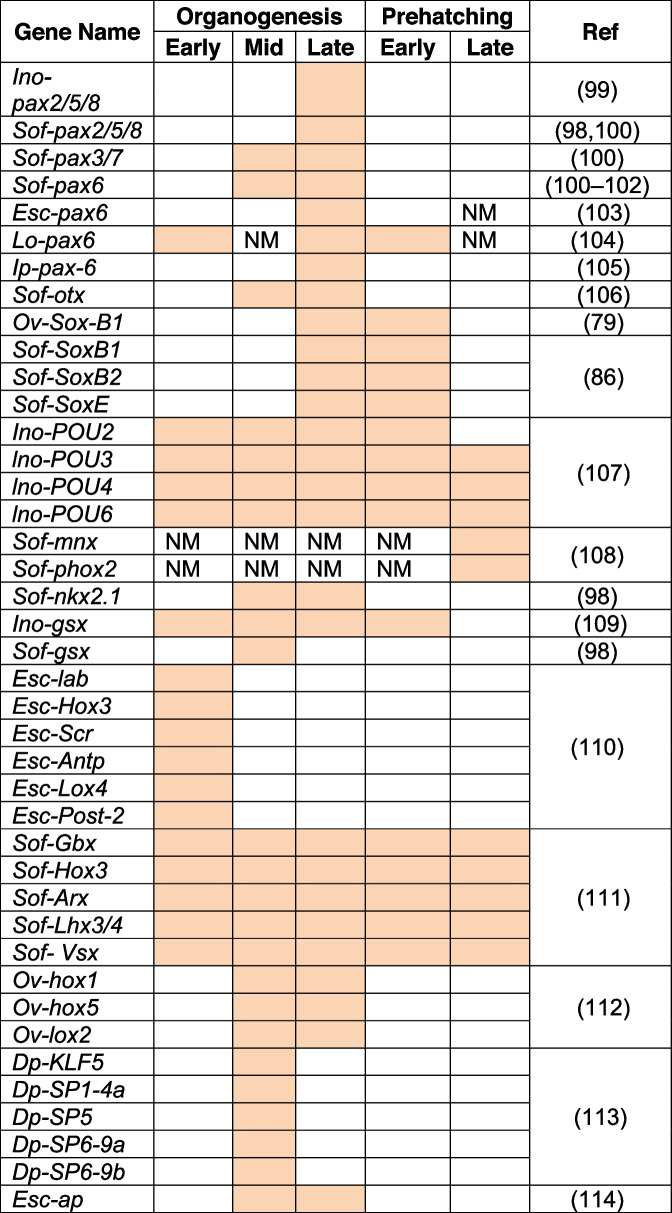
Temporal expression of the transcription factors in the CNS

The table summarises the temporal expression pattern of the transcription factors studied on cephalopod species. This table provides information about the phases in which the spatial expression analysis (displayed in Table 2) was carried out. The expression intensity of the genes is indicated by color: Color - Expression, No Color – No Expression, NM - Not mentioned in the paper. Abbreviations of the studied species: Dp: *Doryteuthis pealeii*; Esc: *Euprymna scolopes*; Ino: *Idiosepius notoides*; Ip: *Idiosepius paradoxus*; Lo: *Loligo opalescens*; Ov: *Octopus vulgaris*; Sof: *Sepia officinalis* [99], [98, 100], [100], [100–102], [103], [104], [105], [106], [79], [86], [107], [108], [98], [109], [98], [110], [111], [112], [113], [114]

Among the intrinsic factors, the paired-box and homeodomain families of transcription factors (TFs) are the most extensively studied gene families in coleoid cephalopods [100, 110–112, 115].

Within the paired-box genes, *Pax6* seems to be a prominent TF involved in the development of the visual and nervous systems in coleoid cephalopods, similar to vertebrates and *Drosophila* [100, 116, 117]. It is predominantly expressed anteriorly in the cerebral cord (later on SEM) and optic cords (later on PEM) [100]*.* Furthermore, it is known in vertebrates that *Pax6* negatively regulates *Shh* and is involved in the specification of dorsal identity in the developing nervous system [118–122]. In *S. officinalis*, it has been shown that the expression patterns of *Pax6* and *Shh* do not overlap since *Shh* is expressed at the cord borders, whereas *Pax6* is expressed in the entire cerebral and optic cords [101]. This could be an indication that *Pax6* and *Shh* also interact with each other in a regulatory way in cephalopods.

Homeodomain genes are known to play a crucial role in the formation and patterning of the central nervous system [123]. Although initial studies found the HOX cluster to be in different scaffolds [10, 124], more recent assemblies found that genes of the HOX cluster are organised on the same chromosome, albeit over a larger genic distance [125]. As expected, their expression marks different brain regions along the A-P and D-V axis, but there seem to be differences between cephalopods and between stages (Table 2, 3) [110, 112, 126]. For the moment, it is still difficult to distil a clear picture, and a clear definition of orthologs and a more systematic approach will be needed before we can conclude whether the observed differences among species are an indication of evolutionary and developmental differences among coleoid cephalopods.

### Extrinsic factors

Even though there have been a few studies exploring the involvement of extrinsic factors in coleoid cephalopod embryogenesis (Notch signalling in eye development [84], Hedgehog signalling in the mantle and its coexpression with Pax6 [101, 127], Wnt Pathway in the cephalopod lens [128], Hedgehog, BMP and Wnt Pathways in the limb [129]), only the Notch signalling pathway has clearly been implemented in (retinal) neurogenesis [97]. Notch signalling is known to maintain neural progenitor identity and regulate cell cycle and differentiation in vertebrates and *Drosophila*. Inhibition of Notch signalling using small molecule inhibitors like DAPT in the squid retina leads to premature cell cycle exit, failure to differentiate into a photoreceptor cell and disorganisation of the retinal layers [84]. In the retina, neuro-epithelial progenitors have to make a binary choice to generate photoreceptor cells that start expressing *EphR*, or supporting cells that express *SoxB1*. Upon Notch inhibition by DAPT, *EphR* expression was increased at the expense of *SoxB1,* suggesting a fate switch towards photoreceptors. The mature photoreceptor marker *rhodopsin* was never expressed, indicating a block of cell differentiation. Notch signalling thus acts as the regulator of the cell cycle exit, differentiation and cell fate determination and conserves the progenitor identity in the squid retina [97]. To our knowledge, it is still unclear whether Notch plays similar roles in neurogenesis in the lateral lips. Also, for other morphogen pathways, no information is yet available on the role extrinsic factors play in controlling neurogenesis or neural migration in the brain.

## Molecularly defined neural cell types

**Fig. 4:**
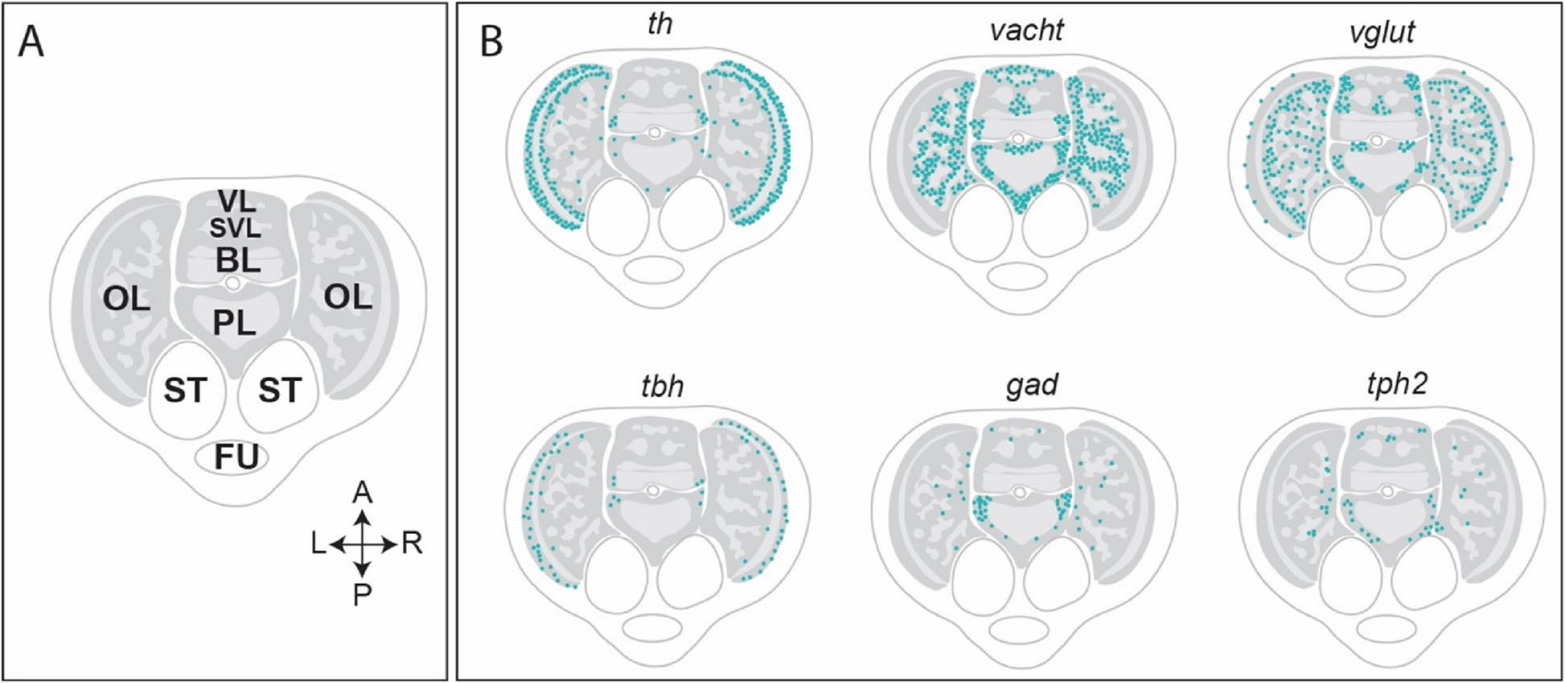
Neurotransmitters in the brain of the cephalopod hatchling. **A** Schematic depiction of a horizontal section through a brain of the hatchling. **B** Spatial depiction of gene expression of tyrosine hydroxylase (*th*) for dopaminergic neurons, vesicular acetylcholine transporter (*vacht*) for cholinergic neurons, vesicular glutamate transporter (*vglut*) for glutamatergic neurons, tyramine beta-hydroxylase (*tbh*) for octopaminergic neurons, glutamate decarboxylase (*gad*) for GABAergic neurons and tryptophan hydroxylase 2 (*tph2*) for serotonergic neurons. Abbreviations: BL: basal lobe; FU: funnel; OL: optic lobe; PL: pedal lobe; ST: statocyst; SVL: subvertical lobe; VL: vertical lobe. Reproduced from Styfhals et al., 2022 [132]

Neural cell subtypes are defined by morphological, molecular and functional characteristics [130]. While adult neural cell morphologies have been extensively documented [5, 131], for embryonic neuronal cell types in coleoid cephalopods the current characterisation relies mainly on the expression of markers for gene expression, including those indicative of neurotransmitter and -peptide usage. In the studied coleoid cephalopod hatchlings, the most prevalent neurotransmitters present in CNS are glutamate, dopamine and acetylcholine. GABA, serotonin and octopamine-producing cells have also been identified, but they appear less in the cephalopod CNS (Fig. 4) [132, 133].

Glutamate is the predominant neurotransmitter, exhibiting a widespread occurrence throughout the entire brain of the hatchling *Octopus vulgaris* and pre-hatchling *Loligo vulgaris*. Expression of the glutamate marker, *vglut* (vesicular glutamate transporter), was observed from the outer granular layer in the optic lobe all the way to the basal lobe (Fig. 4) [132, 133]. Acetylcholine is the second most prominent neurotransmitter, as evidenced by the expression pattern of *vacht* (vesicular acetylcholine transporter), the corresponding marker. In *O. vulgaris*, this expression spanned the whole brain with the exception of the optic lobe's outer granular layer and, to a more limited extent, in the SEM (Fig. 4) [132]. Conversely, *th* (tyrosine hydroxylase), the marker for dopaminergic cells, displayed robust expression in the optic lobe cortex and, to a lesser extent, in the medulla and central brain (Fig. 4) [132]. A dual-transmitter cell type, expressing both dopaminergic and glutamatergic markers, was predominantly situated in the inner granular layer of the optic lobe. A dual dopaminergic and glutamatergic cell type is also observed in the brains of *Drosophila* larvae and vertebrate embryos [132, 134].

The cells expressing other cephalopod neurotransmitters appear in smaller populations. A lower number of serotonergic cells, marked by the sodium-dependent serotonin transporter *sert* and tryptophan hydroxylase 2 (*tph2*), are found in the optic lobe medulla and throughout the central brain which is in line with the embryonic appearance of serotonin (5-HT) in cephalopods [132, 133, 135, 136]. GABAergic neurons represented only a small population of cells spread over different regions of the central brain. In *O. vulgaris*, the expression of *gad* (glutamate decarboxylase) indicates the presence of clusters of GABAergic neurons in the SUB. Additionally, GABAergic neurons were observed in the optic lobe medulla [132, 133]. Octopaminergic neurons expressing *tbh* (tyramine B-hydroxylase) reside in the outer granule layers of the optic lobes in hatchlings and juvenile octopuses, which suggests that cell types in the brain of the hatchling remain present throughout life [137]. Indeed, Songco-Casey et al. discovered a laminated pattern of different cell types throughout the juvenile optic lobe cortex. Several had strong molecular similarities with cell types already present at hatching in *Octopus vulgaris*. Besides octopaminergic cells, dopaminergic, cholinergic as well as glutamatergic and dual dop/glut cell types were present, suggesting that the optic lobe cellular build-up and cell types seem largely conserved at the molecular level, although the repertoire might still expand after hatching [137]. For a more extensive review of the cephalopod optic lobe cell types and function, we refer readers to [138].

Besides using neurotransmitters, it has become clear that cephalopod brains, like many invertebrates, make use of neuropeptides for wireless neurotransmission and neuromodulation. Neuropeptidergic cell types seem prevalent throughout the brain, and the neuropeptide repertoire seems extended [139, 140]. The most studied neuropeptide is FMRFamide, which appears restricted to the palliovisceral cord in early embryonic development in *Idiosepius notoides* [141]. During mid organogenesis, it is found in the middle and posterior SUB, the optic lobes and the posterior basal as well as superior buccal lobes in *Octopus vulgaris* and *Argonauta hians* and in the superior and inferior buccal lobes in *Idiosepius notoides* [136, 141]. Furthermore, *fmrf* expression was observed in the SEM and distributed throughout the optic lobes in *Loligo vulgaris* [133].

Whereas the picture of neuronal diversity is gaining molecular clarity, the types of glial cells remained less studied. In cephalopods, glial cells in the brain have been suggested to contribute to the blood-brain barrier, and phagocytosis of apoptotic cells [142, 143]. Based on recent transcriptomic analysis, three glial subtypes were identified in brain of *O. vulgaris* hatchling, localised mainly in the neuropil tissue, but also in between neurons and in an ependymal-like layer surrounding the brain [132]. One of these subtypes displayed the presence of the neurotransmitter GABA, distinguished by the expression of *gat1* (GABA transporter 1), while the other subtypes did not display neurotransmitter- or peptide characteristics. However, all glial cells exhibited elevated expression levels of *gs2* (glutamine synthetase 2) and *eaat1* (excitatory amino acid transporter 1), both involved in glutamate clearance [132]. This aligns with the expression patterns observed in glial cells in *Drosophila* [144, 145]. In contrast to mammals, where the number of glial cells generally surpasses that of neurons, the brain of the *O. vulgaris* hatchling exhibits a different ratio, with approximately 10% of all brain cells being glial cells, which is similar to other invertebrate brains [132, 146].

In conclusion, the single-cell atlases of coleoid cephalopods have played a crucial role in elucidating the molecular identities of neural cell subtypes. The observed array of neurotransmitters underscores the intricate and diverse nature of neural cell subtypes in these species, shedding light on the complexity of their neural circuitry. Moreover, a parallel characterisation in the juvenile *Octopus bimaculoides* corroborates these findings and indicates that neuronal diversity still increases after hatching [137].

## Conclusions and future directions

The molecular and cell biological study of neurogenesis, neural migration and patterning in coleoid cephalopods is still in its infancy. Recent studies have revealed that mechanisms known from vertebrate model species, such as the use of pro-neural transcription factors, long-distance neuronal migration, and interkinetic nuclear migration, have also been observed in cephalopods [79, 97]. What remains unclear is the cellular organisation of the periocular neurogenic niche and what factors steer the spatial and temporal patterning. One might expect that neurogenic progenitors generate intermediate progenitors as a means to increase neuronal output, but evidence is still lacking. The picture that emerges from the spatial expression of intrinsic transcription factors is still fragmentary, and focused on a single species, time point or tissue.

The fact that neurons migrate long distances seems to indicate that extrinsic signalling molecules and guidance cues play a role, but these are still unknown. Furthermore, knowledge on other neurogenic zones present in the cephalopod body remains very limited. Whether neurogenesis in the developing arm, or stellate and other ganglia present in the mantle or gastrointestinal tract follow a similar temporospatial patterning is still a mystery.

Many of these aspects will become more clear once a more concerted comparative effort can be made using next-generation sequencing methods and molecular tools that have become available to the cephalopod field [147]. A comprehensive approach that combines single-cell RNA sequencing (scRNA-seq) and *in situ* hybridisation or spatial transcriptomics in combination with morphological technologies into 2D and 3D browsable atlases might spur new hypotheses [58]. Now that novel methods have been established [148, 149], functional analysis of the transcription factors driving neurogenesis by genome editing will bring new insights into their roles in cell type specification.

Overall, our current knowledge about the cellular and molecular mechanisms involved in coleoid cephalopod neurogenesis barely scratched the surface of how these incredible animals evolved their way of generating neurons and developing the largest invertebrate nervous system. With this review, we aimed to bring together the available knowledge on this topic and raise questions that can help the cephalopod developmental neurobiology field.

## Data Availability

Not applicable.

## Abbreviations

A: Anterior
*A. hians*: *Argonauta hians*
ar: Arm
asc: Achaete-Scute
ascl1: Achaete-Scute homolog 1
ATZ: Anterior Transition Zone
bHLH: Basic Helix-Loop-Helix transcription factors
BL: Basal Lobe
BLC: Basal Lobe Complex
BrLC: Brachial Lobe Complex
CC: Cerebral Cord
CFDA-SE: Carboxyfluorescein Diacetate Succinimidyl Ester
CNS: Central Nervous System
D: Dorsal
*D. pealeii* (Dp): *Doryteuthis pealeii*
*Drosophila*: *Drosophila melanogaster*
*E. scolopes* (Esc): *Euprymna scolopes*
eaat1: Excitatory Amino Acid Transporter 1
elav: Embryonic Lethal, Abnormal Vision
es: Esophagus
ey: Eye
fu: Funnel
gad: Glutamate Decarboxylase
gat1: GABA Transporter 1
gs2: Glutamine Synthetase 2
*I. notoides* (Ino): *Idiosepius notoides*
*I. paradoxus* (Ip): *Idiosepius paradoxus*
IGL: Inner Granular Layer
IKNM: Interkinetic Nuclear Migration
ISH: *in situ* Hybridization
L: Left
*L. opalescens* (Lo): *Loligo opalescens*
*L. vulgaris*: *Loligo vulgaris*
LL: Lateral Lips
MCLC: Magnocellular Lobe Complex
med: Medulla
mo: Mouth
neuroD: Neuron Differentiation Marker
neurog: Neurogenin
NM: Not Mentioned
*O. berrima*: *Octopus berrima*
*O. bimaculoides*: *Octopus bimaculoides*
*O. vulgaris* (Ov): *Octopus vulgaris*
OC: Optic Cord
OGL: Outer Granular Layer
OL: Optic Lobe
OTC: Optic Tract Complex
P: Posterior
PC: Pedal Cord
pcna: Proliferating Cell Nuclear Antigen
PEM: Periesophageal Mass
PL: Pedal Lobe
pl: Plexiform Layer
PLC: Pedal Lobe Complex
PNS: Peripheral Nervous System
PTZ: Posterior Transition Zone
PVC: Palliovisceral Cord
PVLC: Palliovisceral Lobe Complex
R: Right; Ref: References
*S. lessoniana*: *Sepioteuthis lessoniana*
*S. officinalis* (Sof): *Sepia officinalis*
scRNA-seq: Single-Cell RNA Sequencing
SEM: Supraesophageal Mass
sert: Sodium-Dependent Serotonin Transporter
si: Sinus Ophthalmicus
ST: Statocyst; SUB: Subesophageal Mass
SVL: Subvertical Lobe
*T. pacificus*: *Todarodes pacificus*
tbh: Tyramine B-Hydroxylase
TFs: Transcription Factors
th: Tyrosine Hydroxylase
tph2: Tryptophan Hydroxylase 2
V: Ventral
vacht: Vesicular Acetylcholine Transporter
vglut: Vesicular Glutamate Transporter
VL: Vertical Lobe
VLC: Vertical Lobe Complex
