# Correction: Embryonic development of a centralised brain in coleoid cephalopods

**DOI:** 10.1186/s13064-024-00196-0

**Published:** 2024-11-04

**Authors:** Ali M. Elagoz, Marie Van Dijck, Mark Lassnig, Eve Seuntjens

**Affiliations:** 1 Laboratory of Developmental Neurobiology, Department of Biology, KU Leuven, Leuven, Belgium; 2 Leuven Brain Institute, KU Leuven, Leuven, Belgium; 3 Leuven Institute for Single Cell Omics, KU Leuven, Leuven, Belgium

**Correction: Neural Dev 19, 8 (2024)**

10.1186/s13064-024-00186-2

Following publication of the original article [1], the authors would like to correct the Fig. 1 caption, Consent for publication and Acknowledgements sections of the article. The updated caption and sections were provided below and the changes have been highlighted in **bold typeface**.

Fig. 1 Neural cell count in the nervous system of Bilateria, Cnidaria and Porifera. The total number of neurons in the nervous system (depending on the size of the nervous system and availability of the data) of Bilateria, Cnidaria and Porifera. Bilateria are divided into Protostomia and Deuterostomia. Urbilateria is the last common ancestor between protostomes and deuterostomes. Cephalopods have the highest number of neurons in their nervous system among protostomes. Their neuronal count is even higher than that of some deuterostomes. The mammalian brain is in a neuronal count range similar to the cephalopod nervous system. Porifera has no nervous system. The neuronal count number representatives for the selected groups: Cephalopoda: [5, 6]; Gastropoda: [13]; Annelida: [14, 15]; Platyhelminthes*: [16, 17]; Nematoda: [18]; Arthropoda*: [19]; Mammalia*: [20, 21]; Teleostei*: [22]; Echinodermata: [23]; Cnidaria: [24]; Porifera: [25]. Asterisk (*) symbolises that the number range indicated is for either the brain or the CNS. **Figure credits: adapted from figure presented by Jessica Stock at the Urchin (DBSUMI) Conference in October 2023**

Consent for publication

**Figure 1 was based on an unpublished Figure made by Jessica Stock and Caroline Albertin**. Figure 2 is reproduced from Deryckere et al., 2021 [79], and Fig. 4 is reproduced from Styfhals et al., 2022 [132], which are open-access articles distributed under the terms of the Creative Commons CC BY license.

Acknowledgements

**The authors thank Jessica Stock and Caroline Albertin for providing inspiration for Fig. 1.** The authors would like to thank the members of the Seuntjens and Arckens labs for critical discussions and Maxence Lanoizelet for proofreading the manuscript.

The original article [1] has been corrected.
